# Being sheltered from a demanding everyday life: experiences of the next of kin to people with dementia attending farm-based daycare

**DOI:** 10.1080/17482631.2021.1959497

**Published:** 2021-08-02

**Authors:** Liv Bjerknes Taranrød, Ingeborg Pedersen, Øyvind Kirkevold, Siren Eriksen

**Affiliations:** a Vestfold Hospital Trust, Norwegian National Advisory Unit on Ageing and Health, Tønsberg, Norway; b Faculty of Medicine, University of Oslo, Oslo, Norway; c Department of Public Health Science, Norwegian University of Life Sciences, Ås, Ås, Norway; d Innlandet Hospital Trust, Centre for Age Related Functional Decline and Diseases (AFS), Hamar, Norway; e Department of Health Sciences, Norwegian University of Science and Technology (NTNU), Gjøvik, Norway; f Department of Bachelor in Nursing, Lovisenberg Diaconal University College, Oslo, Norway

**Keywords:** Family caregiver, next of kin, dementia, daycare service, farm-based daycare, care farming, respite, support, qualitative, interviews

## Abstract

**Purpose:**

Farm-based daycare (FDC) is a type of daycare service for people with dementia. The aim of the present study was to explore the next of kin’s experiences with FDC and how the service may affect their daily life.

**Methods:**

The study has a qualitative, descriptive design. Eight semi-structured interviews with next of kin were conducted. The data were analysed in accordance with content analysis.

**Results:**

We identified three main categories: (1) I am fine when you are fine, (2) Significant aspects of the service at the farm, and (3) FDC as a part of the dementia trajectory. The findings were summarized in one overarching, latent theme: “Being sheltered from a demanding everyday life”.

**Conclusions:**

The findings indicate that next of kin’s experience of respite is closely connected to the well-being of their relatives at the FDC and the quality of the service. FDC provides significant support through a part of the trajectory of dementia. Despite experiencing respite and support, next of kin continue to struggle with ethical and moral decisions about the futures of their relatives with dementia.

## Introduction

Dementia affects cognitive abilities and activities of daily living. The condition not only has an impact on the person with dementia but also affects the next of kin (Livingston et al., 2020). Next of kin caring for people with dementia have an increased risk for burden of care, including reduced physical, mental and social health. They may also experience financial challenges related to care tasks (Adelman et al., 2014; del-Pino-Casado et al., 2018). In Norway, it is a policy that people with dementia should live in their own homes as long as possible with the support of individually tailored services and next of kin contributing to their care (Norwegian Ministry of Health and Care Services, 2015, 2018; World Health Organization, 2017). About 90% of people with dementia in Norway receive help from their next of kin, and the help increases during the course of dementia (Vossius et al., 2015). The municipalities are obliged to provide necessary support such as respite, training and guidance for next of kin (Act 2011–06-24-30 Helse- og omsorgstjenesteloven, 2021). Daycare (DC) services are intended to facilitate meaningful activities in a safe environment and to improve quality of life for people with dementia. In addition, they provide respite and support for next of kin (Du Preez et al., 2018; Norwegian Ministry of Health and Care Services, 2007, 2015). Since 2020, all Norwegian municipalities have been obliged to offer DC services for people with dementia (Act 2011–06-24-30 Helse- og omsorgstjenesteloven, 2021).

A review found that DC creates a break from caregiving tasks and reduces feelings of burden, worry and depression among next of kin (Maffioletti et al., 2019). Further, DC may improve quality of life and have a positive influence on the relationship between the next of kin and the person with dementia, leading to improved cooperation and higher quality of time spent together (Maffioletti et al., 2019). Other studies reported that the content and quality of the DC were important for the next of kin’s experiences of respite, i.e., having confidence in the staff and knowing that their relatives with dementia are being well cared for contributed to their experience of respite (Rokstad et al., 2017; Strandli et al., 2016).

To meet the various needs of people with dementia, the Norwegian dementia plan emphasizes the importance of offering different kinds of daycare (Norwegian Ministry of Health and Care Services, 2015, 2020). Farm-based daycare (FDC) service for people with dementia has been established as one type of DC with a similar purpose, and both offer respite for next of kin (Ibsen et al., 2018; Norwegian Ministry of Health and Care Services, 2015). In Norway, the municipality has the overall responsibility for the quality of the health and social services provided within its jurisdiction (Act 2011–06-24-30 Helse- og omsorgstjenesteloven, 2021). FDCs are a collaboration between municipalities and farmers, the municipalities pay for the services provided at private farms (Ibsen et al., 2018).

FDC has been found to prevent an increase in caregiver burden over time (De Bruin, 2009). A wide range of activities are provided related to farm buildings, gardens, animals, and outdoor areas (Ibsen et al., 2018). Further, the participants at FDC are more physically and socially active, and they spend more time outdoors than those at regular DC (Ellingsen-Dalskau et al., 2020; Finnanger Garshol et al., 2020). However, knowledge about the next of kin’s experiences with FDC is limited. Two interview studies found that FDC appears to promote the health of both the person with dementia and his or her next of kin (Solum Myren et al., 2013; Strandli et al., 2016). Strandli et al. (2016) reported that the staff`s dedication to caregiving and to facilitating individual activities were important for the next of kin’s experience of relief and the safety of their relative. The next of kin’s experiences can provide us with an extended understanding of how FDC might influence their daily lives and which elements of FDC can be important. The present study aimed to explore the next of kin’s experiences with FDC and how the service may affect their daily lives.

## Method

### Study design

The present study has a qualitative, descriptive design. The purpose was to gain an understanding of the lived experience from the person’s point of view. The study is ontological and epistemological based on hermeneutic phenomenology, as operationalized in Kvale and Brinkmann (2009). The data was collected using qualitative, individual interviews about the participants experiences as caregivers to people with dementia attending an FDC. Kvale and Brinkmann base their approach to the qualitative interview on postmodern, pragmatic and hermeneutic philosophies. They emphasize that knowledge in qualitative research is not achieved by following value- and interest-free methods, because human subjectivity plays a non-reducible role in the development of qualitative knowledge. Further, Kvale and Brinkmann underline that the importance of good qualitative research is based on good craftsmanship (Kvale & Brinkmann, 2009, p. 78). Therefore, we have strived to be transparent and analysed the data according to the well-recognized method of content analyis (Graneheim & Lundman, 2004; Lindgren et al., 2020).

### Participants and recruitment

To gain an extended understanding of the participants’ experiences, we included a purposeful sample of next of kin reflecting different sex, ages, and relationships with people with dementia (Patton, 2002). The inclusion criteria were being next of kin to people with dementia attending an FDC and meeting the relative with dementia at least once a week.

A group of eight next of kin, were invited to participate, all agreed to take part in the present study. The participants consisted of four men and four women with age ranging from 45 to 86 years. Four were spouses cohabiting with the person with dementia. Four were sons, daughter and a niece not living with the person with dementia. The relatives with dementia had mild or moderate degree of dementia and attended FDC two to four days a week.

The participants were recruited from seven different farms and regions of Norway through the FDC providers or healthcare personnel in the municipalities. All FDC services took place on farms engaged in agricultural production in suburban or rural areas. Five of the six farms had animals such as sheep, cows, goats, hens, rabbits, dogs, or cats.

The present study is part of the research project *“Farm-based daycare services for people with dementia: quality development through interdisciplinary collaboration”*, a prospective study organized into several qualitative and quantitative sub studies, with a multimethod approach (Eriksen et al., 2019). The participants were unknown for the authors prior to the study.

### Data collection

Individual interviews were conducted between June 2017 and February 2018. Four interviews took place in participants’ homes, two in other appropriate places chosen by the next of kin, and two by phone. The interviews were dialogue-based and supported by an interview guide with open-ended questions. We addressed topics about being a caregiver before and after the relative started at FDC, experience of FDC and reflections on future care situation.

The interviews lasted 25–60 mins; each was conducted by the first author (LBT) and tape-recorded. The interviews were transcribed verbatim by LBT and one research assistant.

### Data analysis

The transcribed interviews were analysed in line with qualitative content analysis by Graneheim and Lundman (2004) and focussed on both manifest and latent levels of content. NVivo 12 Pro (QSR International Pty Ltd, 2020) was used to support the coding and organizing of the data. The analysis was conducted by LTB in cooperation with two of the co-authors (SE, IP), and the process could be described in six stages.

First, each interview was read several times to acquire an overview of the material, and each was identified as a unit of analysis. Second, the text was divided into meaning units, and condensed units were formed. Third, the meaning units were extracted and labelled with codes. Fourth the codes were compared based on differences and similarities and then grouped into seven subcategories. In Stage five, the subcategories were clustered and grouped as three categories. Finally, in Stage six, the categories were summarized and reflected on to reach a latent presentation of the text based on an overall theme. An example of the process is shown in Table I.

**Table I.** Examples of the analysis process

Meaning unit	Condensed meaning unit	Codes	Subcategory	Category
*She gets to help with the farm chores, which she knows about from her childhood. The activities are familiar, but I know that they (the staff) organize tasks that are beneficial. I just know that they are having a good experience and that the tasks they are doing are worthwhile*	The next of kin knows that the staff facilitates tasks that the relative with dementia is familiar with and beneficial.	The staff facilitates the tasks individually.	A dedicated staff	**Significant aspects of the service at the farm**
*It’s not a nursing home service, but there’s a sense of community here, which they contribute to a little bit*	Opposite to day care at nursing home, the FDC do not give an institutional feeling	The farm gives a sense of real life	An atmosphere of real life	

### Pre-understanding

The first author (LBT) who conducted all the interviews, is a registered nurse (RN) with a research interest in the care situation for the next of kin of people with dementia. LBT was interviewed by the fourth author (SE) prior to the study with the aim of creating a conscious relationship with her own pre-understanding. The three co-authors (SE, IP and ØK) are researchers with many years of experience in different aspect of dementia care and dementia care research.

### Ethical aspect

The present study was approved by the Norwegian Centre for Research Data (NSD) (no.49799) and conducted in accordance with the Declaration of Helsinki (World Medical Association, INC, 2004) Before each interview was initiated, the next of kin received oral and written information about the study, research topic and gave written consent. The interviewer emphasized that personal confidentiality would be guaranteed and informed that she is a registered nurse (RN).

## Results

The results can be divided into three main categories with underlying subcategories. First, “I am fine when you are fine” describing the experiences of being a caregiver; second, “Significant aspects of the service at the farm” describing the next of kins’ experiences of FDC; and third “FDC as a part of the dementia trajectory” describing thoughts about the future (Table II).

**Table II.** Theme, main categories, and subcategories

Theme	Being sheltered from a demanding everyday life		
Main Category	**I am fine when you are fine**	**Significant aspects of the service at the farm**	**FDC as a part of the dementia trajectory**
Subcategory	Having time, freedom and fewer worries	A dedicated staff	We reached a point
	Mastery and enjoyment for the person with dementia	An atmosphere of real life	Being safe in the system
			The agonies of choices now and in the future

### I am fine when you are fine

The experiences of being a caregiver for a person with dementia attending FDC could be summed up as “I am fine when you are fine”. This describes important aspects for the experience of respite. Most of the participants experienced FDC as a support service for both the relative with dementia and themselves. FDC positively affected their daily lives and offered them respite. They considered FDC to be a safe place and enjoyable for their relatives with dementia. Several described the service as “utterly invaluable regarding our situation”. The participants highlighted two important perspectives of respite: (1) Having time, freedom, and fewer worries; and (2) Mastery and enjoyment for the person with dementia.

#### Having time, freedom, and fewer worries

On days when their relative attended FDC, the participants had opportunities to do things they otherwise could not. Spouses, in particular, underlined the importance of having time to take care of their own health and to rest and recharge when they felt worn out from caregiving.

It means so much, especially when I start to get tired. Tomorrow he’s going up to the farm. I can go for a walk or take a trip into town because I think it’s sad that he sits alone here at home and sits a lot on the days when he’s not up there. Then there’s not much else to do. (Spouse)

The participants had time to do things at their own pace when their relative was at FDC. Without feeling guilty about leaving the relative with dementia, they could pursue their hobbies and participate in social life. Some of the participants described that they wished they could have more days of respite. Several, especially those who did not live with the relative with dementia, described worrying about their relative’s nutritional status, physical health, and passivity. After their relatives started to attend FDC, these worries eased.

#### Mastery and enjoyment for the person with dementia

The experience of respite was closely connected to knowing that the relatives with dementia enjoyed their time at the FDC. When the person with dementia expressed mastery and enjoyment this eased the burden for their relatives.

They have put up some shelves and tidied up, not big tasks. (Staff member) says that it should not be unmanageable. It must be something they can accomplish. I think this is very well thought through, and when it’s clear, when they are given enough time, they enjoy it. After all, it’s the same for us, too. (Spouse)

The FDC staff organized tailored activities that the relatives with dementia were able to master. A daughter said, “The activities are familiar, but I know that they (the staff) organize beneficial tasks.”

The activities were something to look forward to for the relatives with dementia in their daily lives. One spouse stated, “The best thing for him is that he gets up in the morning, and when I see how good his mood is … he has something to go to”. The relatives with dementia participated in farm activities such as cutting and stacking wood, tending plants in the garden, and caring for animals. They also took part in other activities such as hiking, baking, singing, reading aloud from the newspaper, and other forms of social interaction. The next of kin emphasized physical activities as an important part of the day because such activities helped the relatives with dementia to maintain physical function.

Whether they go skiing in the winter or take a walk, whether they are being in the mountain or pasture, or other activities like baking … there are things that she enjoys and that she thinks are fun to be a part of. Of course, this means a lot to me. I feel reassured when (staff member) is leading the activity. (Son)

The FDC influenced the everyday rhythm of life for the relative with dementia. For example, he or she slept better after a day at the FDC, and this entailed a better night for the next of kin with fewer interruptions. Several next of kin further reported that their relative with dementia was in a better mood and had a more positive attitude towards life after starting at the FDC. These changes were attributed to enjoyable activities, social interaction with other attendees and the staff and animals at the farm, and the opportunity to enjoy the outdoors. The relatives with dementia had expanded their social networks and formed new friendships. Some of the interviewees stated, “Now we have something to share and to talk about”, which affected their relationship positively and improved the care situation.

### Significant aspects of the service at the farm

The participants emphasized that the context of the daycare was important for them to feel fine. When describing the experience of FDC, they attributed two important aspects: (1) A dedicated staff; and (2) A real-life atmosphere.

#### A dedicated staff

The participants outlined the staff’s ability to create an inclusive community where their relatives with dementia could be themselves with their individual resources and challenges.

Especially with the wonderful staff there with the social and inclusive aspects, and they (relatives with dementia) can be themselves. That is worth its weight in gold, both for me and my mum. (Daughter)

It was important that the staff were educated and had experience caring for people with dementia. One spouse said, “It’s the daily care and the staff who show concern, assume responsibility and follow up. I feel as though nothing random happens there”. The staff were able to make individual adaptations to activities based on each participant’s level of function and preferences. The staff met the relatives with respect, dignity, and care, and this was highly significant for the next of kin’s experience of security. In addition, the dialogue with the staff, gaining insights into FDC and being reassured that their relatives were fine were essential.

#### An atmosphere of real life

For the participants it was important that the FDC had an atmosphere of real life opposite to a constructed, institutional life. The participants emphasized that the buildings at the farm have the opposite of an institutional feeling. The outdoor area was described as natural and free. A son described how the context influenced his father: “He is a little freer, you know. It’s a farm, and he can go out in the yard or the garden, listen to the birds and walk over to say hello to the sheep”. Being able to have contact with the animals on the farm was beneficial, as several of the relatives attending the FDC had enjoyed experiences with animals in their younger days. Several of the participants expressed that having contact with the animals helped to make the day better for their relatives at the FDC.

They had some rabbits at the farm, and rabbits are something he grew up with and can relate to. So, we walked right over to the rabbit, and we talked about it and had a conversation about the old days. (Son)

The participants expressed that it was an advantage to have small groups of attendants. This made it possible for the relatives with dementia to have time to talk with each other and experience fellowship with someone in the same situation.

### FDC as a part of the dementia trajectory

When asked about the future, most of the participants reported that FDC was the first municipal service the person with dementia had received. And they realized that FDC was a part of the dementia trajectory and that the person with dementia would need other care services. Three perspectives were highlighted: (1) We reached a point where we needed help; (2) Being safe in the system; and (3) The agonies of choices now and in the future.

#### We reached a point where we needed help

For the participants, daily life gradually changed after their relative had developed symptoms of dementia and had become increasingly passive and less interested in taking part in social activities and performing daily tasks. Due to their relatives’ changes in cognitive function and in other functions needed to conduct activities of daily life, the participants had to monitor daily activities more closely and take over responsibility for tasks that the relative with dementia used to do. Most of the participants stated that they found their life circumstances to be challenging. They experienced both grief and worry. A son stated, “It’s no fun to see a person you love sitting inside and being obviously unhappy about it, when you know she used to enjoy being active and spending time outdoors”. Several participants said that their patience was continually put to the test:

Sometimes I feel a little irritated, but then I look at him and get a bad conscience. After all, he can’t help it […]. Not all the days are bad, but now and then things go a bit wrong’ (Spouse).

The participants used different strategies, such as humour, to manage difficult situations; as one spouse said, “We don’t make a big deal out of it; we make the best of it”.

The participants explained that they had reached a point where they had to ask the municipal health services for measures to relieve their burden. In agreement with the relative with dementia, the participants established contact with the healthcare service in the municipality. When the need for services was assessed, the person with dementia was offered a place in FDC.

#### Being safe in the system

For many of the participants, FDC was the first service offered for their relatives with dementia. The participants understood that the progression of dementia and new needs for additional or different measures and support would arise at a certain point. The participants descried that it was important to establish contact with the healthcare service. All participants described the first contact with the healthcare system as a positive experience.

They felt that the concerns and needs of their relative with dementia were taken seriously and the relative with dementia was offered a place in FDC. A spouse commented, “I think to myself that I’m so happy he attends FDC, and I’m pleased that he is in the system”.

From the very first meeting with the FDC staff, the participant felt welcome and received good information about the programme. They describe that both the relatives with dementia and themselves were included in the community the farm.

Several FDCs held regular meetings with the next of kin together with personnel from the healthcare service in the municipality. However, most of the communication with the FDC took place by text messages, telephone calls, emails when considered necessary, or a chat when the relative was picked up at his or her home in the morning. In addition, some of the FDCs used notebooks, monthly newsletters or arranged social events. The participants expressed that the staff at the FDC was caring and supportive about their situation. The staff had close contact with the healthcare system in the municipality and conveyed the needs of individual participants when needed.

#### The agonies of choices now and in the future

The participants expressed a strong desire for the relative with dementia to live as independently as possible, both now and in the future. Furthermore, they thought about the difficult decisions they would have to make about their relative with dementia in the near future. However, receiving FDC service was a way of preparing for the days to come. A son said, “One of the reasons we wanted the municipality involved was that we could discuss measures and the future with experts in the field. This lays the foundation for adding more services when he gradually becomes worse”.

The participants were concerned about their obligations to help and support their relative throughout the course of dementia. A niece stated, “I couldn’t stand to see everything fall apart, to put it one way. There’s no human dignity in that. We have a responsibility as a family”. The participants described challenging discussions in which family members had conflicting ideas about the needs of the relative and when he or she should be moved to a nursing home.

My son says: now you are just pushing him (the father) away. Then I say, yes, but he understands this himself. He knows he’s on the list. He has been informed of this, and he agrees. We are not both going to get sick. (Spouse)

Most of the participants considered FDC as a service that could potentially postpone nursing home placement.

(…) If she had not had the service at the farm or similar service (…) it will have meant that she probably (…) we have had to find other solutions in relation to the living situation. I think this (FDC) contributes to her being able to live at home. (Son)

The participants also highlighted concerns about the relatives with dementias ´ability to understand their situations and make choices for themselves. A son stated,

I think the matter of consent is a challenge. We have not taken his right to consent from him. Consent in relation to adding new things. I’m thinking of the ethical aspects. I absolutely want to contribute and help my father. He has helped me a lot in my life.

### Overall interpretations: “being sheltered from a demanding everyday life”

In the categories presented above, being a next of kin to a person with dementia is described as demanding and as often causing major changes in life for both parties. The participants reached a point where they needed help, and FDC service was experienced as an important form of support and respite that positively influenced daily life for them as well as for their relative with dementia. This positive experience was strongly connected to the tailored and meaningful activities in natural settings created for the attendees by the staff. Being a next of kin to a person with dementia could be described as “being outdoors in rough weather”. FDC was an important part of the dementia trajectory. Having the relative with dementia attending FDC created an important break or shelter from their daily struggle. Therefore, the latent meaning of our findings could be summed up as: “being sheltered from a demanding everyday life”.

## Discussion

The present study aimed to explore the next of kin’s experiences with the service at the farm and how FDC may affect their daily life. The participants were most concerned with the well-being of the persons with dementia. When the next of kin knew that their relatives had a good time at the FDC, they experienced a break from the daily worries and could enjoy time and freedom to follow their interests or meet their own needs. This gave the next of kin a possibility to re-energize and recover from caregiving. Other studies have also reported that FDC may promote personal time, fewer feelings of guilt and an experience of respite for the next of kin (De Bruin et al., 2015; Solum Myren et al., 2013; Strandli et al., 2016). In our study the next of kin reported that relatives with dementia slept better after attending FDC. This, in turn, resulted in more-restful nights with fewer interruptions for the participants. Tretteteig et al. (2017) study of next of kin to people with dementia attending a regular daycare also noted this.

A recent study concerning next of kin of people with dementia attending a FDC reported that the perceived burden of care is dependent on the living situation (Taranrød et al., 2020). The study reported that spouses living with a person with dementia attending FDC experienced a significantly higher burden than next of kin who did not live with their relative (Taranrød et al., 2020). In our study, several participants emphasized a need for more service than the relatives with dementia were currently being offered by the municipality. In Norway, the municipalities have the responsibility to support and tailor interventions to next of kin who experience burden of care (Helse- og omsorgstjenesteloven 2011; Norwegian Directorate of Health, 2017). Enjoyable and meaningful activities which contribute to a feeling of mastery for the person with dementia is the basis for daycare services (Norwegian Ministry of Health and Care Services, 2020). Despite significant effort by the authorities to increase the number of daycare services, there is still a lack of available services to meet the next of kin’s needs for relief (Granbo et al., 2019; Norwegian Directorate of Health, 2019; Norwegian Ministry of Health and Care Services, 2015, 2020).

For next of kin in general, it is not enough that their relatives attend a daycare service; the context of the service and how the service is organized are just as important (Tretteteig et al., 2015). The next of kin may hesitate to use services that are not perceived as beneficial for the person with dementia (Neville et al., 2015). Our participants described FDC as positive for their relatives with dementia and highlighted the atmosphere of “real life” surrounded by farm buildings and outdoor areas. “Real life” was described in contrast to “institutional life” since most regular daycare in Norway are situated in institutions, such as nursing homes (Norwegian Directorate of Health, 2019). Our findings correspond with those of other studies (Solum Myren et al., 2017; Strandli et al., 2016). The farm environment provided opportunities for a variety of useful activities that promoted participation and a feeling of freedom for the person with dementia (Strandli et al., 2016). Solum Myren et al. (2017) also described the farm environment (including staff) as a context that enables attendees to participate more in everyday activities compared to ordinary daycare. To some people with dementia and their next of kin, it is difficult to accept traditional service offered for people with dementia, and a “real-life” setting could make this more acceptable (Strandli et al., 2016). Stephan et al. (2018) found that the attitudes and beliefs informal caregivers had towards formal care were predominantly reticent or negative as most services were currently judged to be too focused on the disease rather than on the person with the disease; additionally, they felt that the psychological and social needs of their relative were often not appropriately considered (Stephan et al., 2018). The Norwegian National guidelines for dementia state that all care and services should be person-centred (Norwegian Directorate of Health, 2017a). Brooker (2014) and Kitwood (1997) specified that an environment that supports positive interaction contributes to supportive social psychology, which is an important factor in person-centred care. From the perspective of our participants, the staff at the farms seemed to use the environment to promote individual care for the persons attending FDC.

Our participants perceived the staff as important for promoting their relatives’ well-being, and they expressed confidence in the staff. The staffs’ expertise in dementia care, their engagement, and their skills in adjusting service to the needs and resources of the persons with dementia were highly valued. The study by Schols and van der Schriek-van Meel (2006) found that next of kin were more satisfied with the service at FDC than that at regular daycare. To our participants, it was important that the staff managed to create an environment for social inclusion and to facilitate meaningful activities for their relative. This was also seen by Stephan et al. (2018), who found that the competencies of the health and social care professionals, their dementia-specific knowledge and their awareness of each person with dementia and his or her social competencies were important for the next of kin. Other studies have shown that the staff and the farm environment may promote connection and autonomy for the attendees (Ellingsen-Dalskau et al., 2020; Hemingway et al., 2016; Ibsen & Eriksen, 2020) and that well-organized daycare provides support and enriches everyday life for people with dementia as well as their next of kin (Gústavsdóttir, 2011). In addition, next of kin to persons with dementia attending FDC described that meaningful days at the farm and a sense of fellowship were perceived as promoting health both for the person with dementia and the next of kin, who experienced the service as a relief (Solum Myren et al., 2013; Strandli et al., 2016).

Caring for a person with dementia could be described as a dynamic process, meaning that the care responsibilities as well as the next of kin’s experiences change as the dementia progresses (Montgomery & Kosloski, 2009). The participants highlighted the experience of being in the middle of the dementia trajectory where FDC was one, and often the first, service encountered along the journey. The period before receiving FDC was characterized by exhaustion, grief and worries about the situation. At the same time, they experienced substantial responsibilities and multiple roles in the care of their relative with dementia. Our findings align with the findings of earlier research describing the next of kin’s experiences of the life situation before contacting the healthcare system (Moholt et al., 2018; Solum Myren et al., 2013; Tretteteig et al., 2017; Vossius et al., 2015). In the early stage of the disease, next of kin may not experience a need for help and may not identify themselves as “carers” (Stephan et al., 2018). Our participants described having reached a point where help from the healthcare system became a necessity and, in agreement with their relative with dementia, they contacted healthcare services.

Throughout the trajectory of dementia, both the person with dementia and the next of kin’s situation may change considerably due to social isolation and the loss of a social network in addition to increased stress, strain, depression, and other health-related problems associated with caregiving (Brodaty & Donkin, 2009; Lethin et al., 2020). Support from healthcare services is crucial and may reduce the next of kin’s feelings of strain and burden. Stephan et al. (2018) found that next of kin expect to share the responsibility of caring for the person with dementia with healthcare personnel and to receive help for making joint decisions regarding the care. Our participants experienced having rapport and helpful dialogues with the FDC staff. They felt safe knowing that they had someone with whom to share the responsibility of care. A recent study of next of kin of people with dementia attending an FDC found that social support positively affected the quality of life and burden of care for the next of kin (Taranrød et al., 2020), and in a review by Williams et al. (2019), multicomponent interventions including learning coping strategies and getting emotional support were found necessary to reduce caregiver burden.

Being a next of kin may elicit feelings including commitment and responsibility (Davies & Nolan, 2004). Our participants were in a position where, in the near future, they would have to make difficult decisions about choices for a higher level of care for their relatives with dementia. Making such choices may generate guilt and distress for those who must make them (Davies & Nolan, 2004; Larsen et al., 2020) and seems to be agonizing for the participants in relation to several ethical dilemmas. In a recent qualitative review of spouses’ experiences (Egilstrod et al., 2019), lack of control and uncertainty about the future were particularly pronounced. It is important that healthcare personnel are aware of the next of kin’s struggles to cope with the situation and that they facilitate adequate support to the next of kin (Larsen et al., 2020).

### Methodological considerations

In their own words, the participants in the present study described their experiences of FDC and how the service affected their daily lives. Lincoln and Guba (2000) highlighted five areas of importance for quality in qualitative studies: credibility, dependability, confirmability, transferability, and authenticity. To ensure that we have addressed these five areas, we have attempted to describe the process openly and reflexively, presenting each step of the research in detail. The interviews were analysed on both manifest and latent levels of the content (Graneheim & Lundman, 2004). We are aware that there may be more than one correct interpretation of the transcribed interviews. The data are from only eight participants, and this may have influenced the results and, thereby, the transferability of our findings to other populations may be limited. By contrast, the sample represents different FDCs and regions of Norway. The participants are diverse in regard to age, sex and whether the participant lived with the relative with dementia or not. Thus, we believe the findings elucidate important experiences of next of kin that may be transferable to other next of kin of persons with dementia.

## Conclusions

The main finding of this study was that the next of kin’s experience of respite was closely connected to the well-being of their relative at the FDC and the quality and content of the service. The next of kin faced the agony of making choices about how to care for their relatives with dementia, and they seemed willing to take responsibility with support from the healthcare system. Our findings underpin the importance of having someone with whom to share the responsibilities of care and having a good quality healthcare service that supports the next of kin along the trajectory of the progression of dementia. FDC can be a important “shelter” for next of kin and offer good days for people with dementia during this trajectory.
